# Unlocking the sealed entrance—exploring left coronary treatment options: a case report

**DOI:** 10.1093/ehjcr/ytaf663

**Published:** 2025-12-20

**Authors:** Yoshihiro Imai, Atsushi Hirohata, Hirofumi Nakajima

**Affiliations:** Department of Cardiology, Nakashima Hospital, 122 Tamachi, Tsuyama, Okayama 7080052, Japan; Department of Cardiology, The Sakakibara Heart Institute of Okayama, 2-5-1 Nakai-cho, Kita-ku, Okayama 7000804, Japan; Department of Internal Medicine, Nakashima Hospital, 122 Tamachi, Tsuyama, Okayama 7080052, Japan

**Keywords:** Case report, Left coronary atresia, Congenital coronary artery disease, Reconstruction

## Abstract

**Background:**

Left main coronary artery (LMCA) atresia is a rare anomaly characterized by the absence of the left coronary ostium, a blind-ending left main trunk, and retrograde collateral flow from the right coronary artery. Clinical manifestations may include angina, syncope, dyspnoea, sudden death, or failure to thrive. Information on the treatment of this anomaly is extremely limited, and the long-term outcomes of conventional coronary artery bypass grafting in young patients remain uncertain.

**Case summary:**

A 37-year-old woman presented with recurrent syncope during adolescence and later developed chest pain and cardiac arrest during childbirth. Coronary angiography confirmed LMCA atresia, yet repeated stress perfusion tests consistently showed no ischaemia. Considering the limited negative predictive value of stress testing and insights from guidelines addressing other congenital coronary anomalies, surgical reconstruction was performed using an external iliac artery (EIA) autograft directly anastomosed from the aorta to the LMCA. The harvest site was repaired with a vascular prosthesis. The patient recovered uneventfully and has remained stable under medical therapy.

**Discussion:**

This case represents the first reported surgical reconstruction of LMCA atresia using an EIA autograft. It highlights the limitations of ischaemia testing in congenital coronary anomalies, the potential advantages of reconstruction over conventional bypass surgery in the setting of competitive native flow, and the importance of individualized, thoughtful decision-making. External iliac artery autograft reconstruction may offer a promising alternative for patients with this rare condition.

Learning points

- Coronary artery atresia is a potential underlying cause of sudden cardiac death.
- Risk stratification is challenging due to infrequent ischaemia detection and the probable low negative predictive value of stress tests.
- Individualized, rational surgical reconstruction, including external iliac artery autograft, may offer a durable and effective alternative to conventional bypass grafting.

## Introduction

Left main coronary artery (LMCA) atresia is a rare coronary anomaly in which there is no left coronary ostium, the proximal left main trunk ends blindly, and blood flows from the right coronary artery to the left in retrograde fashion via collateral arteries. Usually patients present with angina, syncope, dyspnoea, sudden death, and or failure to thrive. Information about this uncommon anomaly is extremely limited, especially in its treatment. In past reports, coronary artery bypass grafting (CABG) was performed as a general treatment, however, continued uncertainty remains regarding long-term results among those of a young age. Also, there is a report of a teenage patient in which the blood flow of an internal mammary artery graft was insufficient, and the patient had to rely on the other graft, a saphenous vein graft to the obtuse marginal artery.^1^

## Summary figure

**Table:** 

Timeline	Events
16-year-old	A 16-year-old female had undergone an exercise myocardial perfusion single photon emission computed tomography (SPECT) for repeated loss of consciousness with exercise stress electrocardiogram showing remarkable ST segment depression, demonstrated no evidence of ischaemia, which resulted in simple follow-up observation.
23-year-old	Repeated chest pains led to detailed examinations via coronary computed tomography and coronary angiography which indicated a left coronary ostial atresia and resulted in conservative treatment using a beta-blocker. Re-evaluated exercise myocardial perfusion SPECT showed no change in ischaemia.
25-year-old	An exercise myocardial perfusion SPECT indicated negative for ischaemia.
26-year-old	During childbirth, she went into cardiac arrest and was successfully resuscitated.
27-year-old	A fourth exercise myocardial perfusion SPECT resulted as negative for ischaemia again.
37-year-old	Surgical reconstruction was performed using the left external iliac artery autograft.
30-day follow-up	Follow-up at 30 days showed patient with no symptoms, with being maintained on aspirin and a beta-blocker.

## Case presentation

**Figure 1:**
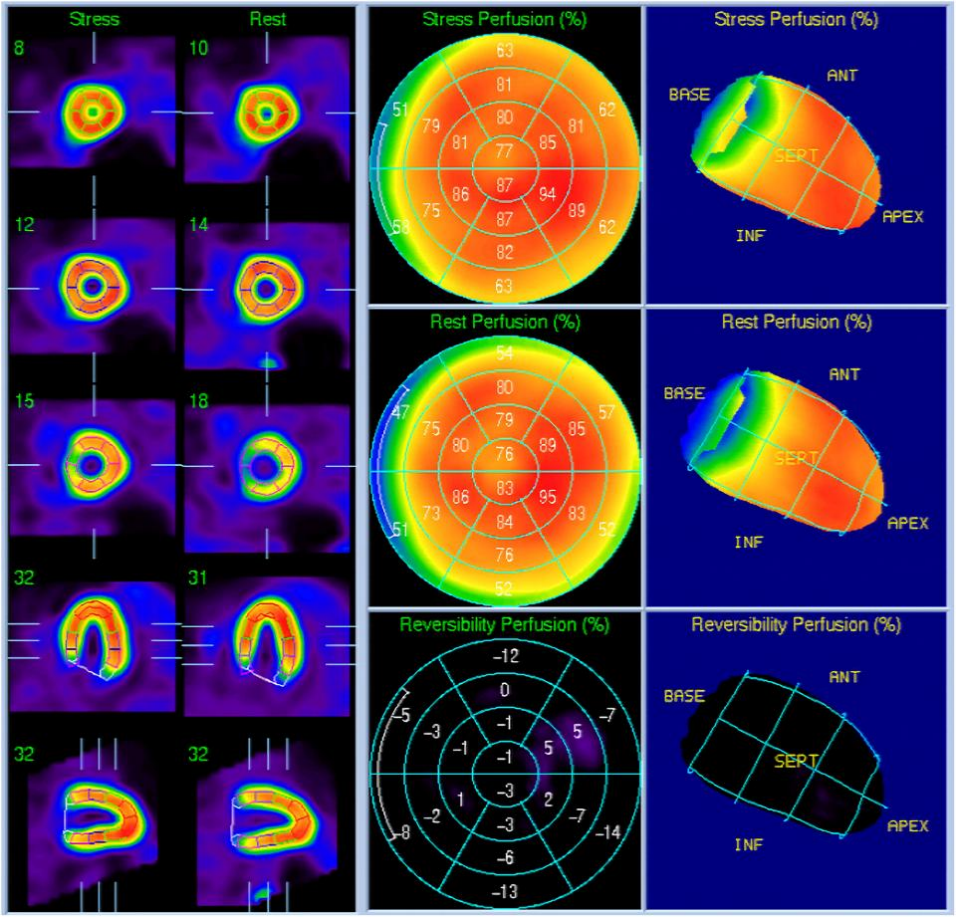
A pre-operative stress myocardial scintigraphy image. There were no findings of perfusion abnormality, transient ischaemic left ventricular dilatation, or transient left ventricular dysfunction, suggesting the absence of myocardial ischaemia, with end-diastolic volumes and ejection fractions of 62 mL/70% at rest and 54 mL/67% under stress, respectively.

A 37-year-old female had undergone an exercise myocardial perfusion single photon emission computed tomography (SPECT) with an exercise stress electrocardiogram showing remarkable ST segment depression at the age of 16 for repeated loss of consciousness during her high school athletic activities, which demonstrated no evidence of ischaemia and resulted in simple follow-up observation (*Figure 1* and *Videos 1* and *2*).

**Figure 2:**
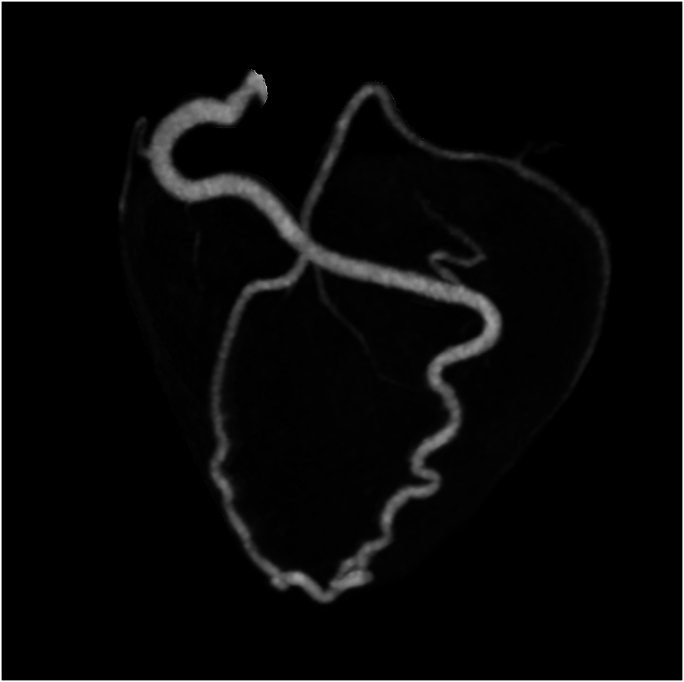
A pre-operative maximum intensity projection image of a coronary computed tomography. The right coronary artery and LAD are connected directly in the distal segment.

**Figure 3:**
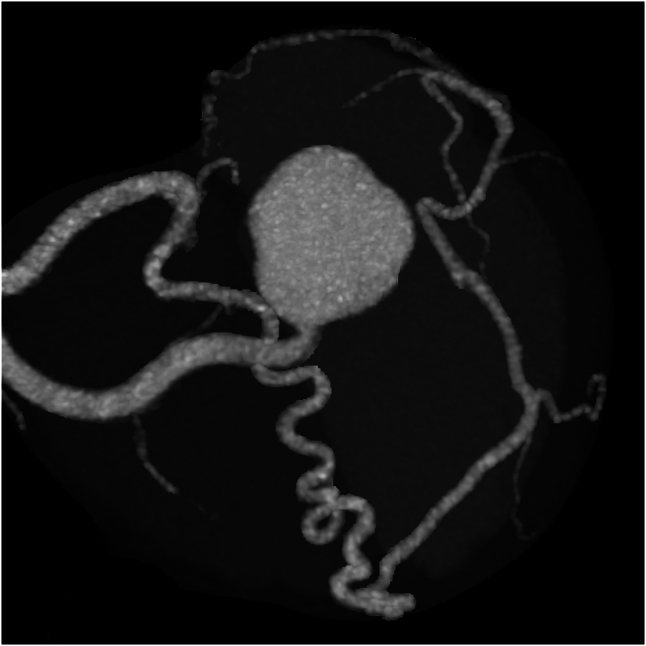
A pre-operative cranial view of the Valsalva sinus. No communication between the left sinus of Valsalva and the left main trunk is observed, indication of morphology consistent with atresia of the left coronary artery (LCA) ostium.

**Figure 4:**
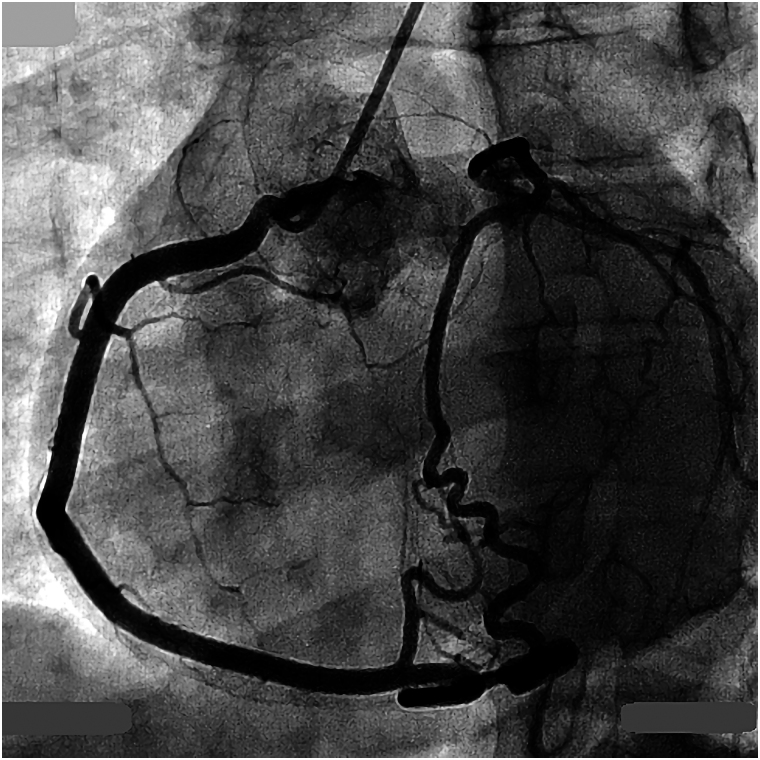
A pre-operative coronary angiography. Contrast injection into the right coronary artery opacified the left coronary artery with revealing a slight separation between the left sinus of Valsalva and the expected location of the left main coronary artery.

Repeated chest pains, 7 years later at the age of 23, led to detailed examinations via coronary computed tomography (CT) and coronary angiography which indicated a left coronary ostial atresia and resulted in conservative treatment using a beta-blocker (*Figures 2–4*). Yet when re-evaluated by exercise myocardial perfusion SPECT, there was still no ischaemia detected. During childbirth, 11 years ago at the age of 26, she went into cardiac arrest and was successfully resuscitated, despite having had a pre-pregnancy exercise myocardial perfusion SPECT which also showed no ischaemia present. This event led her to a fourth exercise myocardial perfusion SPECT, which once again resulted as negative for ischaemia. Based on the significant ST segment depression accompanied by chest pain observed on the exercise electrocardiogram, the presence of myocardial ischaemia was considered evident (Supplementary materials, *Figures S1* and *S2*). Given the patient’s history of cardiac arrest, the risk associated with additional stress testing was deemed extremely high, and no further stress tests were performed.

It is already known that a major challenge in risk stratification for patients with certain congenital coronary artery diseases, such as anomalous aortic origin of coronary artery (AAOCA), is the infrequent identification of ischaemia and the probable low negative predictive value of a stress test.^2^ As a result, current ESC and ACC/AHA guidelines recommend surgical intervention for AAOCA with high-risk anatomy, such as an intramural course or orifice anomalies including a slit-like orifice, acute-angle take-off, or an origin more than 1 cm above the sinotubular junction, regardless of the identified ischaemia.^3,4^ In addition, unroofing or reimplantation is recommended as a revascularization procedure as opposed to CABG because of the high probability of early graft failure.^5^ This date can be useful in the selection of a treatment plan for similar congenital coronary artery diseases such as in this patient’s case.

**Figure 5:**
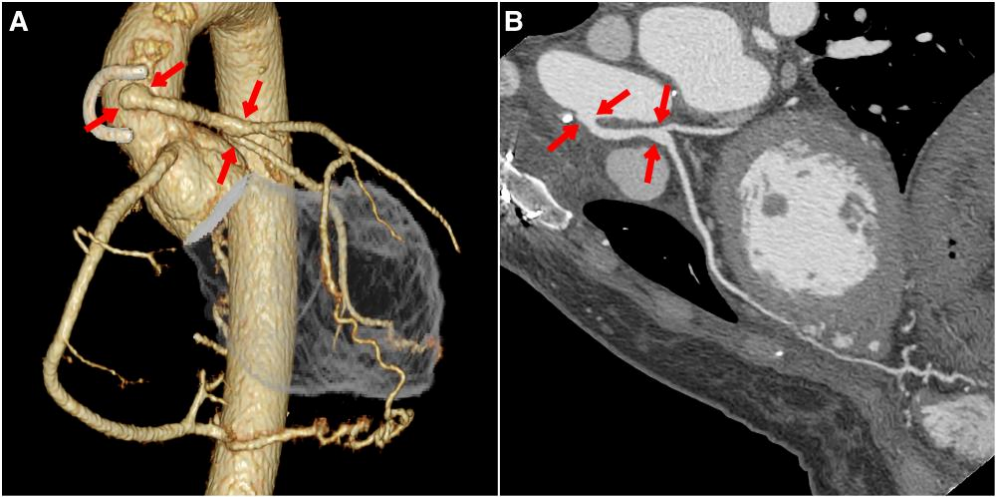
Post-operative coronary computed tomography images of volume rendering (*A*) and multiplanar reconstruction (*B*). The harvested external iliac artery is anastomosed as a free graft from the aorta to the proximal portion of the left coronary artery. The anastomotic sites are indicated by arrows.

Taking all these indications into account and following consultations with the patient, a decision to treat via surgical reconstruction was taken and then subsequently performed using the left external iliac artery (EIA) instead of CABG. The reconstruction was accomplished by directly anastomosing the harvested EIA from the aorta to the region corresponding to the LMCA (*Figure 5*). The harvesting site of the EIA was replaced with a vascular prosthesis. The rationale behind this unique treatment choice was based on a careful analysis of the specific pathology. Unlike typical coronary artery disease with stenosis, LMCA atresia presents as an absence of the left coronary ostium, requiring a complete reconstruction of the proximal LMCA. Given the high-flow nature of the native collateral circulation and the absence of any distal stenoses, the use of the internal mammary artery, gastroepiploic artery, or radial artery was considered to carry a high risk of early occlusion due to competitive native coronary flow. Therefore, the EIA was chosen as the most appropriate option, given its larger calibre and favourable potential for long-term patency. Furthermore, since the patient is a young female without any atherosclerotic risk factors, the long-term outcome of the vascular prosthesis used at the EIA harvesting site is expected to be favourable. The procedure resulted in an optimal outcome with no indication of post-surgery symptoms with continued treatment using aspirin and beta-blocker.

## Discussion

This case required definitive revascularization due to severe clinical course and the unequivocal presence of myocardial ischaemia. Surgical reconstruction was deemed more favourable than conventional CABG, and the EIA autograft was considered the optimal graft for this patient. This case illustrates why direct reconstruction with an EIA autograft was preferred over other graft options, reflecting a patient-specific, pathology-driven decision aimed at achieving durable and reliable myocardial perfusion.

To the best of our knowledge, this is the very first reported experience of LMCA atresia successfully treated with reconstructive surgery using an EIA autograft. This case highlights the limitations of stress testing in risk assessment and emphasizes the challenges of ischaemia detection, the rationale for choosing an EIA autograft over conventional surgical procedures, and the post-operative success of this alternative strategy. Optimal management of this rare anomaly remains controversial, particularly in young patients, and this report illustrates how careful and rational decision-making, including deviation from CABG in favour of direct aorto-LMCA reconstruction using an EIA autograft and consideration of guidelines for anatomically distinct conditions such as AAOCA, can provide valuable clinical insights for managing complex and potentially life-threatening coronary anomalies.

## Lead author biography


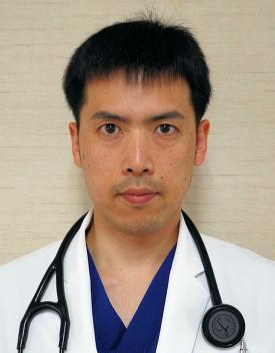


Yoshihiro Imai, MD, is Chief of Doctors at Nakashima Hospital, Japan. He trained in internal medicine and cardiology at specialized institutions, including The Sakakibara Heart Institute of Okayama and Hokkaido Ohno Memorial Hospital. He is a board-certified member of the Japanese Society of Internal Medicine, a Certified Cardiovascular Specialist of the Japanese Circulation Society, and a Certified Interventional Cardiologist of the Japanese Association of Cardiovascular Intervention and Therapeutics. His academic interests include rare coronary anomalies, complex coronary lesions, and innovative interventional strategies, and he has published clinical case reports and studies in these fields.

## Supplementary Material

ytaf663_Supplementary_Data

## Data Availability

All data underlying this case report are included within the manuscript. No additional datasets were generated or analysed for this study.
