# Supplementary figures and images for: Unlocking the sealed entrance—exploring left coronary treatment options: a case report
Source: Eur Heart J Case Rep. 2025 Dec 20;10(1):ytaf663. doi: 10.1093/ehjcr/ytaf663 (PMC12963720; doi:10.1093/ehjcr/ytaf663)

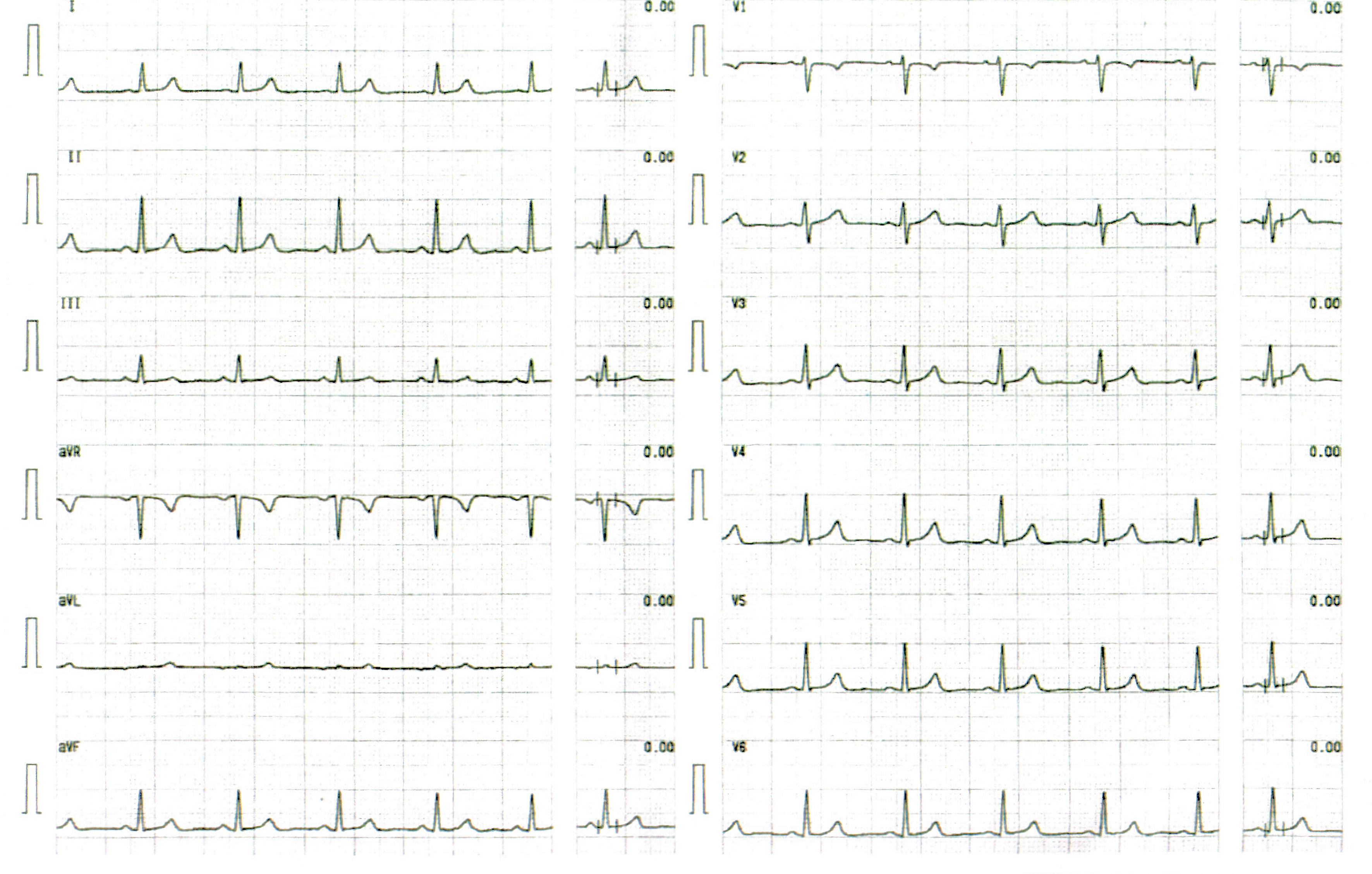

Supplement: ytaf663_Supplementary_Data [file ytaf663_Supplementary_Data.zip › Figure S1.tif]

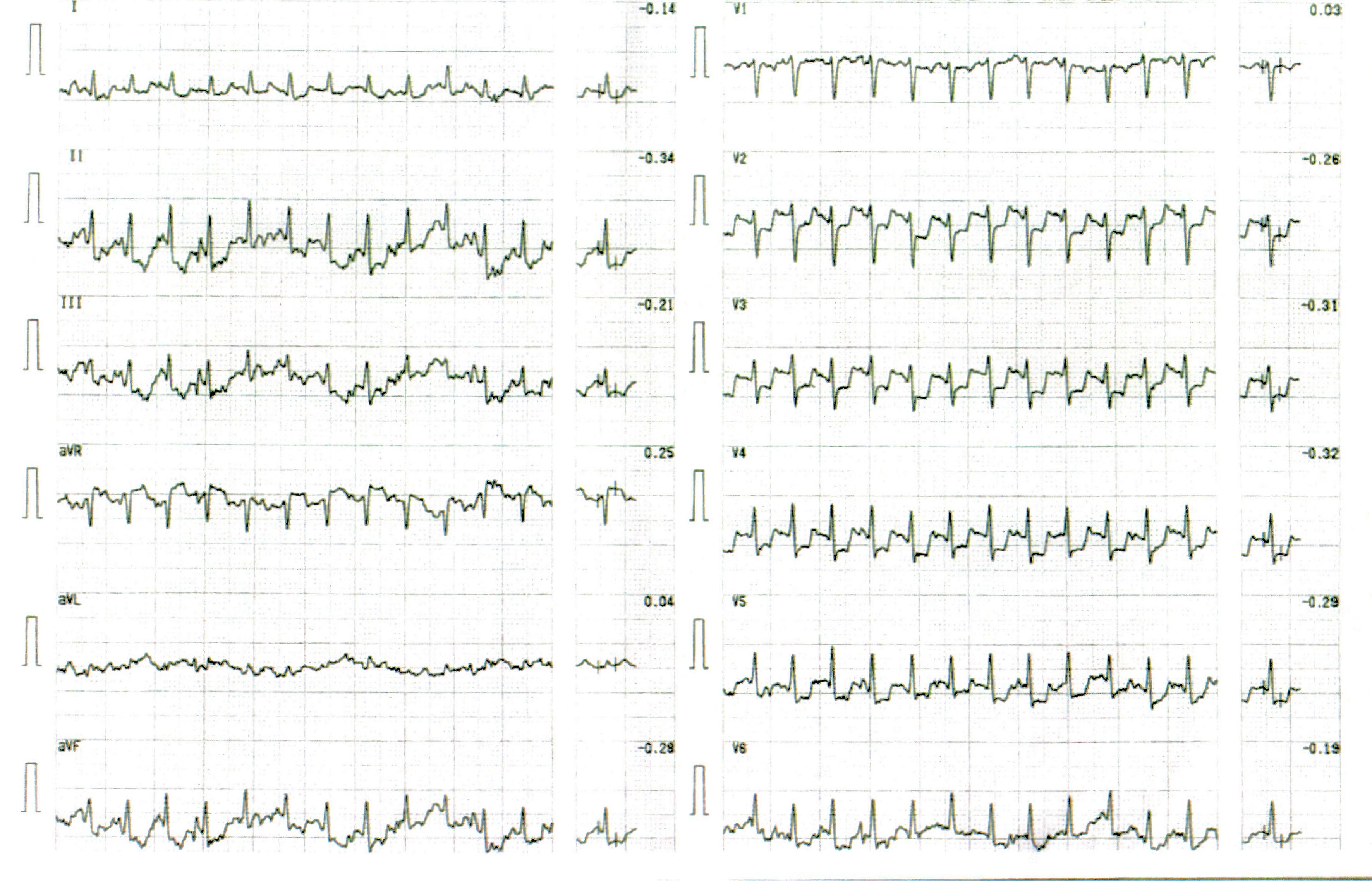

Supplement: ytaf663_Supplementary_Data [file ytaf663_Supplementary_Data.zip › Figure S2.tif]
